# The mechanism of interactions between tea polyphenols and porcine pancreatic alpha‐amylase: Analysis by inhibition kinetics, fluorescence quenching, differential scanning calorimetry and isothermal titration calorimetry

**DOI:** 10.1002/mnfr.201700324

**Published:** 2017-08-23

**Authors:** Lijun Sun, Michael J. Gidley, Frederick J. Warren

**Affiliations:** ^1^ Centre for Nutrition and Food Sciences, ARC Centre of Excellence in Plant Cell Walls, Queensland Alliance for Agriculture and Food Innovation The University of Queensland Queensland Australia; ^2^ Quadram Institute Norwich Research Park Norwich UK

**Keywords:** α‐Amylase, Differential scanning calorimetry, Fluorescence quenching, Interaction mechanism, Isothermal titration calorimetry, Tea polyphenols

## Abstract

**Scope:**

This study aims to use a combination of biochemical and biophysical methods to derive greater mechanistic understanding of the interactions between tea polyphenols and porcine pancreatic α‐amylase (PPA).

**Methods and results:**

The interaction mechanism was studied through fluorescence quenching (FQ), differential scanning calorimetry (DSC) and isothermal titration calorimetry (ITC) and compared with inhibition kinetics. The results showed that a higher quenching effect of polyphenols corresponded to a stronger inhibitory activity against PPA. The red‐shift of maximum emission wavelength of PPA bound with some polyphenols indicated a potential structural unfolding of PPA. This was also suggested by the decreased thermostability of PPA with these polyphenols in DSC thermograms. Through thermodynamic binding analysis of ITC and inhibition kinetics, the equilibrium of competitive inhibition was shown to result from the binding of particularly galloylated polyphenols with specific sites on PPA. There were positive linear correlations between the reciprocal of competitive inhibition constant (1/*K*_ic_), quenching constant (*K*_FQ_) and binding constant (*K*_itc_).

**Conclusion:**

The combination of inhibition kinetics, FQ, DSC and ITC can reasonably characterize the interactions between tea polyphenols and PPA. The galloyl moiety is an important group in catechins and theaflavins in terms of binding with and inhibiting the activity of PPA.

**Abbreviations**
BTE: black tea extracts
C: catechin
DSC: differential scanning calorimetry
EC: epicatechin
ECG: epicatechingallate
EGC: epigallocatechin
EGCG: epigallocatechingallate
FQ: fluorescence quenching
GTE: green tea extracts
ITC: isothermal titration calorimetry
*K*_ic_: competitive inhibition constant
*K*_iu_: uncompetitve inhibition constant
*K*_FQ_: fluorescence quenching constant
*K*_itc_: binding constant
OTE: oolong tea extracts
PPA: porcine pancreatic α‐amylase
TA: tannic acid
TF: theaflavin
TF1: theaflavin‐3’‐gallate
TF2: theaflavin‐3, 3’‐digallate

## 1 Introduction

Dietary phenolic compounds have been suggested as potential alternatives to medicines for controlling and treating type II diabetes, a chronic disease caused by reduced insulin sensitivity, as they have inhibitory effects on α‐amylase 1, 2. Alpha‐amylase is present in saliva and secreted by the pancreas into the small intestine. It catalyses the digestion of dietary starch to maltooligosaccharides. These maltooligosaccharides are further degraded to glucose, which is absorbed into the blood stream, triggering glycaemic and insulaemic responses 3. The inhibitory activities of polyphenols are highly dependent on their molecular structures 4, and the inhibition of α‐amylase results from the interactions and/or binding of polyphenols with the enzyme. Hydrogen bonding between the hydroxyl groups and the active site of the enzyme, as well as hydrophobic interactions between the aromatic moieties of polyphenols and enzyme are considered to be the prime forces that drive the interactions (binding) 5.

Previously, the interactions between polyphenols and α‐amylase have been characterized by determination of the half inhibition concentration (IC_50_), kinetics of inhibition, fluorescence quenching (FQ) and docking analysis 6. The IC_50_ indicates the strength of inhibition, i.e. lower IC_50_ value means higher inhibitory activity. From the kinetics of inhibition, the inhibition type can be identified, and competitive inhibition constant (*K*_ic_) and uncompetitive inhibition constant (*K*_iu_) are obtained 7. Furthermore, the fluorescence quenching constant (*K*_FQ_) indicating the binding affinity of polyphenols to the enzyme is obtained from the quenching effects of polyphenols on the intrinsic fluorescence of tryptophan side chains within the structure of α‐amylase 8. However, the correlation between *K*_FQ_ and 1/*K*_ic_ needs further study, as both constants suggest the binding of polyphenols with the enzyme 6. Tea polyphenols, including (‐)‐epicatechingallate (ECG), (‐)‐epigallocatechingallate (EGCG), theaflavin‐3, 3′‐digallate (TF2), theaflavin‐3′‐gallate (TF1) and theaflavin (TF) have all been reported to have α‐amylase inhibition activity in vitro 8, 9, 10, 11. However, the precise mechanism of the inhibition of α‐amylase by tea polyphenols has not been elucidated in terms of binding interactions between polyphenols and enzyme.

Differential scanning calorimetry (DSC) can be applied to monitor phase and conformational transitions through measurement of specific heat capacity as a function of temperature for a sample 12. It offers an objective and comprehensive way for evaluating the thermal stability of proteins 13. It has been reported that polyphenol interactions may change the thermostability of proteins 14, 15. Therefore, DSC can be used to study the effect of tea polyphenols on the thermostability of α‐amylase. Isothermal titration calorimetry (ITC) permits the determination of binding enthalpy and binding constant of the reaction between a macromolecule and a ligand 16. This technique has been successfully applied to determine the binding constants of oligomericellagitannins with bovine serum albumin 17, 18, 19. To our knowledge, the combination of DSC and ITC has not been previously applied to study the interactions of polyphenols and porcine pancreatic α‐amylase (PPA).

In our previous study, the kinetics of inhibition (IC_50_, *K*_ic_ and *K*_iu_) of PPA by a range of tea polyphenols were systematically studied 6. In this study, FQ, DSC and ITC were combined together with the kinetics of inhibition to elucidate the mechanism of binding interactions of tea polyphenols and PPA. In this approach, 1/*K*_ic_, *K*_FQ_ and *K*_itc_ are compared and correlated to analyze the relationships between PPA inhibition and binding behavior. The role of the galloyl moiety in binding of catechins and theaflavins with PPA is highlighted.

## 2 Materials and methods

### 2.1 Materials and chemicals

Aqueous extracts of various teas (TE), green (GTE), black (BTE) and oolong (OTE) were prepared as described 6. PPA (EC 3.2.1.1 A6255) was purchased from Sigma‐Aldrich Co. Ltd. (St. Louis, US). Pure phenolic compounds including tannic acid (TA), (+)‐catechin (C), (−)‐epicatechin (EC), (−)‐epigallocatechin (EGC), (−)‐epigallocatechingallate (EGCG), (−)‐epicatechingallate (ECG), theaflavin (TF), theaflavin‐3′‐gallate (TF1) and theaflavin‐3, 3′‐digallate (TF2) were obtained from Chengdu Biopurify Phytochemicals Ltd. (Chengdu, China). Other chemicals were of analytical grade.

### 2.2 Kinetics of inhibition

The kinetics of inhibition of PPA by TEs and tea polyphenols were as reported in our previous research 6. In brief, 20 mg/mL normal maize starch was prepared in PBS buffer and cooked at 90°C for 20 min before dilution to serial concentrations (1.25–15 mg/mL). Then for each starch concentration, 50 μL of TEs and pure polyphenols with different concentrations were pre‐incubated with 50 μL of 7.65 U/mL PPA solution (in PBS buffer) at 4°C for 20 min, followed by the addition of 4 mL of cooked starch, and the digestion process was carried out at 37°C. The initial reaction velocity was determined using the PAHBAH method described in our previous study 6. To calculate the competitive inhibition constant, *K*_ic_ and uncompetitve inhibition constant, *K*_iu_, Dixon (1) and Cornish–Bowden (2) equations were applied as follows 7:

(1) v=V max aKm1+iK ic +a

(2) va=V max Km1+iK ic +a1+iK ic

where *v* is the initial reaction velocity determined in the experiment, *V*_max_ is the maximum initial reaction velocity, *a* is the concentration of starch, *K_m_* is the Michaelis constant, *i* is the concentration of inhibitor, *K*_ic_ is the competitive inhibition constant and *K*_iu_ is the uncompetitive inhibition constant.

### 2.3 Fluorescence quenching

Fluorescence spectra of PPA in the absence and presence of TEs and pure polyphenols were recorded by using a Shimadzu^®^ spectrofluorimeter (RF‐5301 PC, Tokyo, Japan) according to a previously reported method 20 with some modifications. Briefly, three TEs were dissolved in PBS buffer and diluted to 0.25, 0.5, 1.0, 2.0, 3.0, and 4.0 mg/mL. Concentration series of pure polyphenols were prepared at 0.025, 0.05, 0.1, 0.2, 0.4, 0.8 mg/mL, respectively. Polyphenol solution (0.2 mL) (or TE solution) was added to a tube containing 3 mL of 0.07 mg/mL PPA solution and mixed thoroughly, followed by incubation at 4°C for 30 min. The control comprised 3 mL of the enzyme solution plus 0.2 mL of PBS buffer. After incubation, each sample solution was transferred into a quartz cuvette pre‐washed using distilled water and sample solution in sequence. Then, the cuvette was loaded into a small cell and the fluorescence spectra were recorded immediately at fast speed and low sensitivity with the excitation wavelength (λ_ex_) set as 282 nm and the emission λ_em_ recorded from 300 to 500 nm. Both the slit widths were 10 nm.

Fluorescence quenching is described by the Stern–Volmer equation:

(3) $$\frac{F_{0}}{F}=\;1+k_{q}\tau_{0}\;\left[Q\right]=\;1+K_{FQ}\left[Q\right]$$

where *F_0_* and *F* are the fluorescence intensity in the absence and presence of the quencher (TE or tea polyphenol), respectively; *k_q_* is the bimolecular quenching constant, *τ*_0_ is the lifetime of the fluorophore. For α‐amylase, the *τ*_0_ is 2.97 ns 21; [*Q*] is the concentration of the quencher; *K*_FQ_ is the fluorescence quenching constant.

Usually, a linear Stern–Volmer plot indicates that there is a single class of fluorophore in the protein interacting with the quencher in the same way and that only one quenching mechanism (dynamic or static) takes place. However, positive deviations for the equation are frequently observed when the quenching extent is large. In this case, the plot of *F_0_/F* against [*Q*] describes an upward curve, concave towards the *y* axis. Commonly, the upward curvature indicates that there are several mechanisms responsible for the quenching effects on fluorophores in protein, or it suggests the existence of a ‘sphere of action’, i.e. apparent static quenching 22. The modified form of the Stern–Volmer equation describing this situation is as follows 23:

(4) $$\frac{F_{0}}{F}=e^{\left(K_{FQ}\left[Q\right]\right)}.$$

### 2.4 Differential scanning calorimetry

The thermostability of the PPA control and that bound with TEs and pure phenolic compounds was studied by use of a differential scanning calorimeter (DSC Q2000, TA^®^ Instrument, New Castle, DE). Solutions of eight individual phenolic compounds (60 mg/mL), concentrations of the three TEs (40, 80 and 120 mg/mL) and 47 mg/mL PPA were prepared in PBS (20% DMSO) buffer. The DSC procedure was performed according to a previous method *^13^* with some modifications: 50 μL of PPA and 50 μL of each polyphenol (or TE) solution were mixed thoroughly in a 1.5 mL microcentrifuge tube. After incubation for 30 min at 4°C, 15 μL of the mixture was pipetted into a TA^®^ Tzero pan and tightly sealed with a Tzero Hermetic lid. 15 mg of each sample solution was loaded. The control was PPA with PBS (20% DMSO) buffer. Thermograms were recorded from 10 to 100°C with 5°C/min heating rate, using an empty pan as the reference. The enthalpy values (*∆H*) were calculated based on the mass of protein in DSC sample pans (J/ g protein).

### 2.5 Isothermal titration calorimetry

A GE^®^ ITC instrument (MicroCal iTC_200_, Stockholm Sweden) was used to determine enthalpy changes associated with polyphenol–PPA interactions at 298 K. In a typical ITC experiment, a polyphenol solution in the injection syringe was titrated into 1.175 mg/mL PPA solution in the sample cell of the calorimeter with stirring at 1000 rpm. All the solutions were prepared in PBS buffer. Each polyphenol solution (10 mg/mL for TEs, ECG, EGCG, TF1 and TF; 5 mg/mL for TA and TF2) was titrated as a sequence of 20 injections. The volume of each injection was 2 μL and the duration of each was 4 s. The time delay between the injections was 150 s. Control experiments included the titration of polyphenol solutions into PBS buffer, the titration of PBS buffer into PPA solution and the titration of PBS buffer into PBS buffer. The latter two control experiments resulted in no measurable enthalpy changes; therefore, they were not considered in the data analysis 19. The control data of polyphenols titrated into PBS buffer was always subtracted from the sample data. The raw data were obtained as a plot of heat flow (μcal s^−1^) against time (min). Then, the data was integrated peak‐by‐peak and normalized using MicroCal Origin (MicroCal Inc.) to obtain a plot of corrected enthalpy change per mole of injection *(∆H* kJ mol^−1^) against molar ratio of polyphenol to PPA (or weight ratio of TEs to PPA). The data obtained were fitted using a single‐site binding model. The equation for this binding model is 24

(5) Qi=nMΔHV021+PnM+1nK itc M−1+PnM+1nK itc M2−4PnM

where *Q_i_* is the total heat released after injection *i*, *V_0_* is the volume of the calorimeter cell, [*M*] is the total concentration of PPA, [*P*] is the total concentration of polyphenols, *n* is the molar ratios of interacting species, *∆H* is the enthalpies, *K*_itc_ is the equilibrium binding constants.

### 2.6 Statistical analysis

The data in this study are expressed as the means of duplicates and analyzed through one‐way analysis of variance (ANOVA) using SPSS 18.0 Statistics (Chicago, USA). The mean values were evaluated by Dunnett's *t*‐test at the 95% significance level (*P *< 0.05). The ITC data were analyzed to obtain the binding parameters and fit standard deviation using MicroCal Origin software.

## 3 Results

### 3.1 Kinetics of inhibition

In our previous study 6, the phenolic constituents of the three TEs were analyzed by UPLC. All TEs contained different contents of catechins, including C, EC, ECG, EGC and EGCG. Both black tea extract (BTE) and oolong tea extract (OTE) contained TF, and only BTE contained TF1 and TF2 6. The inhibitory activities against PPA of pure phenolic compounds were in the order of TF2>TF1≈TA>TF>ECG>EGCG>EC>EGC as indicated by IC_50_ values, which are shown together with values of *K*_ic_ and *K*_iu_ in Supporting Information Table S1 as reported previously 6.

### 3.2 Fluorescence quenching

**Figure 1:**
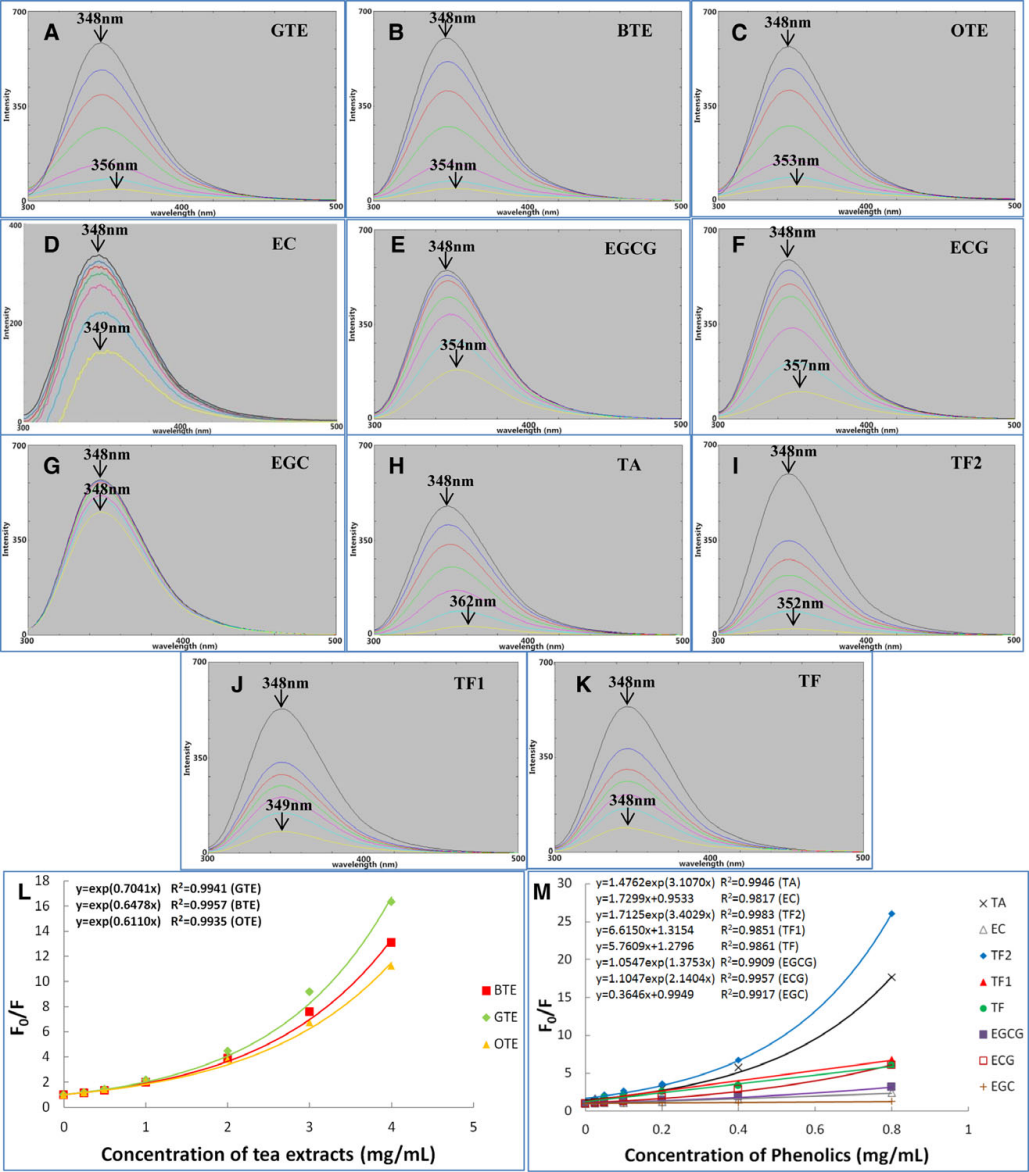
Fluorescence spectra of PPA in the absence (black line) and presence (coloured lines) of GTE (**A**), BTE (**B**), OTE (**C**), EC (**D**), EGCG (**E**), ECG (**F**), EGC (**G**), TA (**H**), TF2 (**I**), TF1 (**J**) and TF (**K**). From top down, the concentrations of three TEs are 0, 0.25, 0.5, 1.0, 2.0, 3.0, 4.0 mg/mL, and the concentrations of eight pure polyphenols are 0, 0.025, 0.05, 0.1, 0.2, 0.4, 0.8 mg/mL. The values labelled in plot (A–K) are the maximum λ_em_ at 0 and highest concentrations of phenolic compounds, respectively; Stern‐Volmer plots for fluorescence quenching of PPA by three TEs (**L**) and eight pure polyphenols (**M**). The equations for EC, TF1, TF and EGC were fitted according to equation (3), and equations for three TEs, TA, TF2, EGCG and ECG were fitted according to equation (4).

The fluorescence intensity of PPA before and after addition of tea polyphenols was determined to investigate the interactions between them. Figure 1A–K shows the fluorescence emission spectra of PPA obtained at *λ*_ex_ = 282 nm with addition of three TEs; (GTE, BTE and OTE) and eight pure phenolic compounds (EGCG, ECG, EGC, EC, TA, TF2, TF1, and TF). In all cases a decrease in the fluorescence intensity, albeit to different extents, was observed from the quenching. Notably, there was a red‐shift of the maximum *λ*_em_ for PPA with three TEs, EGCG, ECG, TA and TF2, while no significant shift was observed for EC, TF1, TF or EGC. The Stern–Volmer plots presented for EC, TF1, TF and EGC showed a linear character, and the plots for TEs, EGCG, ECG, TA and TF2 had an upward curvature, concave toward the *y* axis (Fig. 1L and M). Hence, the original Stern–Volmer Eq. (3) was applied for EGC, EC, TF1 and TF, and the modified Stern–Volmer Eq. (4) was applied for TEs, EGCG, ECG, TA and TF2 to calculate the quenching parameters. The *K*_FQ_ and bimolecular quenching constants (*k_q_*) for TEs and pure polyphenols are summarized in Table 1, in which the respective orders of *K*_FQ_ values for TEs and pure polyphenols are GTE>BTE>OTE and TF2>TA≈TF1>TF>ECG>EGCG>EC. Both these orders are similar to those of the inhibitory activities of these compounds against PPA evaluated by IC_50_ values (Supporting Information Table S1).

**Table 1:** Fluorescence quenching parameters for the interactions of TEs and pure polyphenols with PPA

Phenolic compounds	FQ parameters		
	*K*_FQ_ (M^−1^)	*k*_q_ (10^11^ M^−1^ s^−1^)	Red‐shift of maximum λem (nm)
GTE	0.70^C^ (mL/mg)	2.37^C^ (10^8^ mL mg^−1^ s^−1^)	8^B^
BTE	0.65^B^(mL/mg)	2.18^B^(10^8^ mL mg^−1^ s^−1^)	6^A^
OTE	0.61^A^ (mL/mg)	2.06^A^ (10^8^ mL mg^−1^ s^−1^)	5^A^
EC	273.07^b^	0.92^b^	1^ab^
EGCG	630.4^c^	2.12^c^	6^d^
ECG	946.85^d^	3.19^d^	9^e^
EGC	111.67^a^	0.38^a^	0^a^
TA	5285.60^g^	17.80^g^	14^f^
TF2	11711.81^h^	39.43^h^	4^c^
TF1	4740.24^f^	15.96^f^	1^ab^
TF	3252.03^e^	10.95^e^	0^a^

^*^Different letters in the same column represent significantly different mean values (*P*<0.05).

### 3.3 Differential scanning calorimetry

**Figure 2:**
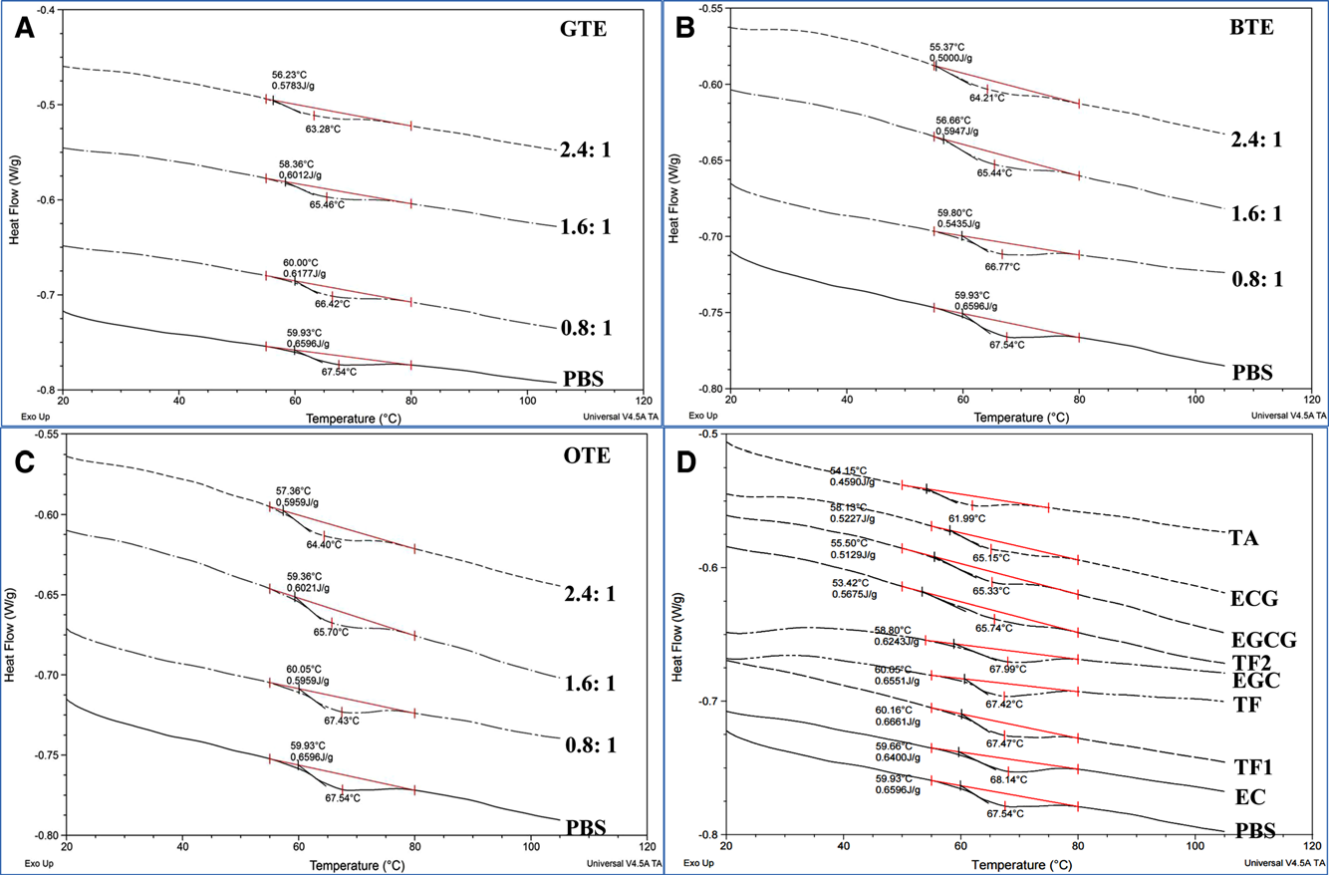
DSC thermograms of PPA treated with GTE (**A**), BTE (**B**), OTE (**C**) and eight pure polyphenols (**D**). The mass ratios of TEs to PPA are 0.8:1, 1.6:1 and 2.4:1, and the concentration of each pure polyphenol used is 60 mg/mL.

DSC was applied to assess interactions with PPA in terms of thermal stability. The effects of TEs and pure polyphenols on DSC characteristics of PPA are presented in Fig. 2. All the PPA–TE (or PPA–phenolic) complexes showed endotherms, meaning no complete denaturation due to polyphenol binding was observed. The DSC thermogram of the PPA control showed a single transition with a peak denaturation temperature (*T_d_*) at 67.54°C and denaturation enthalpy (*∆H*) of 26.91 J/g protein (Table 2). The thermal stability of bound PPA, as reflected by *T_d_*, decreased as a function of the concentration of GTE, reaching 63.28°C at the highest GTE concentration applied (120 mg/mL). The lowest *T_d_* of PPA bound with BTE and OTE were also observed at their respective highest concentrations and determined as 64.21 and 64.40°C, respectively. Generally, the *∆H* of PPA bound with TEs was lowered as well. A lower *∆H* demonstrates that a smaller amount of energy is required to unfold the PPA molecule. Together with the decreased *T_d_* by TEs, this indicates that PPA molecules bound with TEs were less thermally stable than non‐bound PPA. For pure phenolic compounds,the *T_d_* of PPA bound with TA, ECG, EGCG and TF2 decreased by 5.55, 2.39, 2.21 and 1.80°C, respectively, while TF, TF1, EGC and EC had no significant effects on the *T_d_* of PPA. Consistent with the changing tendency of *T_d_*, the *∆H* of PPA bound with TA, ECG, ECCG and TF2 decreased to 18.40, 21.32, 20.39 and 23.15 J/g protein, respectively. However, the *∆H* values for TF, TF1, ECG and EC did not change significantly.

**Table 2:** Denaturation temperature (*T*_d_) and enthalpy (*∆H*) of PPA obtained by DSC thermograms in the absence and presence of TEs and pure polyphenols

Parameters	Phenolic compounds																	
	PBS	TA	ECG	EGCG	TF2	EGC	TF	TF1	EC	GTE			BTE			OTE		
										0.8:1	1.6:1	2.4:1	0.8:1	1.6:1	2.4:1	0.8:1	1.6:1	2.4:1
*T_d_* (°C)	67.54^f^	61.99^a^	65.15^d^	65.33^d^	65.74^d^	67.99^f^	67.42^f^	67.47^f^	68.14^fg^	66.42^e^	65.46^d^	63.28^b^	66.77^e^	65.44^d^	64.21^c^	67.43^f^	65.70^d^	64.40^c^
*∆H* (J/g protein)	26.91^g^	18.40^a^	21.32^c^	20.39^b^	23.15^d^	25.47^f^	26.72^g^	27.17^g^	26.11^fg^	25.20^ef^	24.52^e^	23.59^d^	26.90^g^	24.26^e^	20.40^b^	24.30^e^	24.56^e^	24.30^e^

^*^Different letters in the same line represent significantly different mean values (*P*<0.05).*∆H* was calculated based on the mass of proteins in DSC sample pans.

### 3.4 Isothermal titration calorimetry

**Figure 3:**
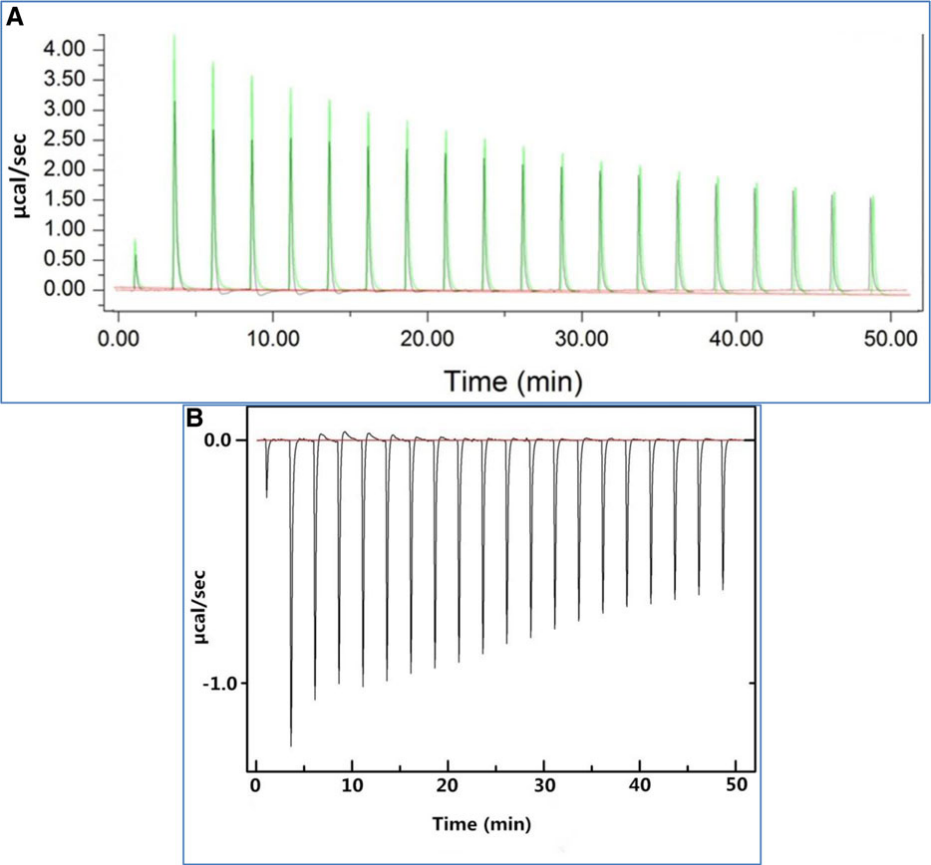
Typical raw (**A**) and corrected (**B**) plots of heat flow against time for titration of TA into PPA. Plot (B) was obtained by subtracting the heat flow of titration of TA into PBS buffer (green line in plot (A)) from the heat flow of titration of TA into PPA solution (black line in plot (A)).

ITC is a powerful technique to analyze the thermodynamics of binding of polyphenols with proteins 18, 25. The interactions of eight phenolic compounds and three tea extracts with PPA were investigated by ITC. Figure 3A shows a typical plot of heat flow against time for titration of tannic acid into PBS buffer (green line) and PPA solution (black line). The positive energy flow for titration of tannic acid into buffer was caused by the heat that was released by the dilution of tannic acid. The corrected heat flow for binding of tannic acid with PPA (Fig. 3B) was obtained by subtracting the dilution heat from the apparent titration heat. The heat flow plots for titration of other polyphenols into PPA solution showed similar dilution responses. For each polyphenol–PPA system studied, an exothermic binding was observed as the corrected heat flow was negative.

**Figure 4:**
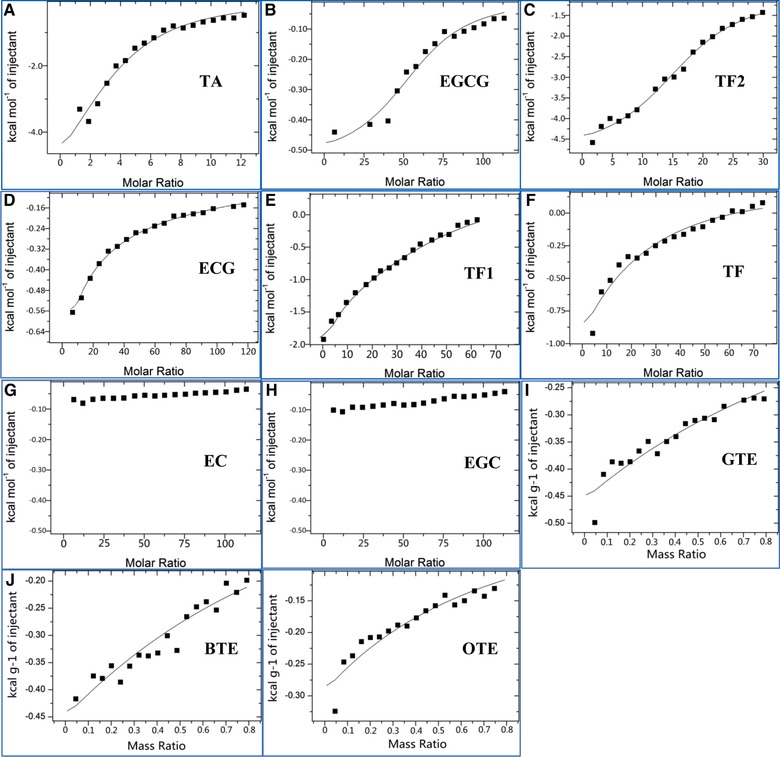
Single‐site biding model fitted to the experimental ITC data for the interaction of TA (**A**), EGCG (**B**), TF2 (**C**), ECG (**D**), TF1 (**E**), TF (**F**), EC (**G**), EGC (**H**), GTE (**I**), BTE (**J**) and OTE (**K**) with PPA. The model could not fit the data for EC and EGC due to the very small level of heat generated during the titration process.

The single‐site binding model that assumes a single set of multiple binding sites 24 was applied to assess the correlation between interaction heat and molar ratio of polyphenol to PPA. The estimated thermodynamic binding parameters are summarized in Table 3. Inspection of Fig. 4 indicates that the single‐site binding model could fit the binding of pure polyphenols better than the three TEs, and the fit standard deviations (the indicator of the goodness of fit) in Table 3 also suggest this. It should be noted that heat was generated during the process of titration of galloylated polyphenols (TA, EGCG, TF2, ECG and TF1) into PPA (Fig. 4A–E), while very little heat was generated during the titration of non‐galloylatedcatechins (EC and EGC) into PPA (Fig. 4G and H). For the pure polyphenols studied, TF2 showed the highest values of *K*_itc_ (11600 M^−1^) and *∆H* (−8790 J mol^−1^), indicating the strongest binding with PPA and the greatest amount of heat released during the binding process. Similarly, EGCG was shown to have the weakest interaction with PPA with the lowest values of *K*_itc_ (482 M^−1^) and *∆H* (−988 J mol^−1^). In addition, TA showed the lowest, and ECG showed the highest stoichiometry (*n*), i.e. the polyphenol to PPA ratio.

**Table 3:** Thermodynamic binding parameters for the interactions of tea polyphenols with PPA fitted by single‐site binding model

Parameters	TA	EGCG	TF2	ECG	TF1	TF	GTE	BTE	OTE
*K*_itc_ (M^−1^)	8740 ± 292^d^	482 ± 106^a^	11600 ± 1750^e^	486 ± 105^a^	5345 ± 1014^c^	1965 ± 553^b^	0.70 ± 0.35 (L·g^−1^)^B^	0.65 ± 0.42 (L·g^−1^)^A^	0.61 ± 0.10 (L·g^−1^)^A^
*∆H* (J·mol^−1^)	−7273 ± 2022^e^	−988 ± 189^a^	−8790 ± 1250^ef^	−1562 ± 569^b^	−5214 ± 1586^cd^	−4256 ± 996^c^	−0.54 ± 0.24 (J·g^−1^)^A^	−0.56 ± 0.32 (J·g^−1^)^A^	−0.56 ± 0.14 (J·g^−1^)^A^
*N*	4 ± 1^a^	52 ± 4^f^	15 ± 1^b^	38 ± 3^e^	17 ± 4^c^	24 ± 6^d^	NA	NA	NA
*∆S* (J·mol^−1^·K^−1^)	6 ± 2^a^	14 ± 3^cd^	5 ± 1^a^	13 ± 1^cd^	10 ± 1^b^	12 ± 5^bc^	0.07 ± 0.01 (J·g^−1^·K^−1^)^A^	0.07 ± 0.01 (J·g^−1^·K^−1^)^A^	0.08 ± 0.01 (J·g^−1^·K^−1^)^A^
SD	30.1	43.6	10.6	4.6	8.9	36.5	359.6	396.4	284.6

^*^Different letters in the same line represent significantly different mean values (*P*<0.05).‘NA’, not available. SD is the standard deviation around fit obtained by MicroCal Origin software.

## 4 Discussion

The difference in fluorescence quenching activities of three TEs indicates that the changes in the micro‐environment of Trp residues caused by interactions of TEs and PPA may depend on the phenolic constituents in TEs. In comparison with the data of PPA inhibition, it was found that a higher quenching effect of polyphenols corresponded to a stronger inhibitory activity.

Fluorescence quenching can be classified into dynamic and static patterns, in which the former results from collisional encounters between fluorophore and quencher, and the latter is caused by formation of a ground state (complex) between the two compounds 26. As mentioned, the upward curve characteristics of Stern–Volmer plots for TEs, TF2, TA, EGCG and ECG suggest that PPA fluorescence was able to be quenched through both dynamic and static mechanisms by these compounds or it might describe the existence of a ‘sphere of action’ (apparent static quenching) 27. On the other hand, the linear characteristics of Stern–Volmer plots for EC, EGC, TF1 and TF mean that only one mechanism (dynamic or static) of quenching occurred for these polyphenols. The bimolecular quenching constant, *k_q_*, which reflects the efficiency of quenching or the availability of quenchers to fluorophores, can be used to determine if the quenching results from complex formation between proteins and quenchers. The *k_q_* is close to 1 × 10^10^ M^−1 ^s^−1^ for the typical dynamic mechanism (collision‐controlled quenching) 26. The *k_q_*values of EC, TF1 and TF are 9‐150‐fold higher than that given in Table 1, suggesting fluorescence quenching of PPA by the three polyphenols involves a static mechanism (complex‐controlled quenching). As fluorescence quenching describes how a quencher affects Trp or its micro‐environment dynamically or statically upon the interactions of the quencher and protein, a higher fluorescence quenching constant suggests stronger affinity of a quencher to protein 27, 28. TF2 and TA showed stronger binding affinity to PPA than the other phenolic compounds in this study due to their higher *K*_FQ_ values (Table 1), presumably due to the large number of potential hydrogen bonds and hydrophobic interactions 29, 30, consistent with the prevalence of the static mechanism in comparison to the dynamic one for PPA fluorescence quenching by TF2 and TA.

**Figure 5:**
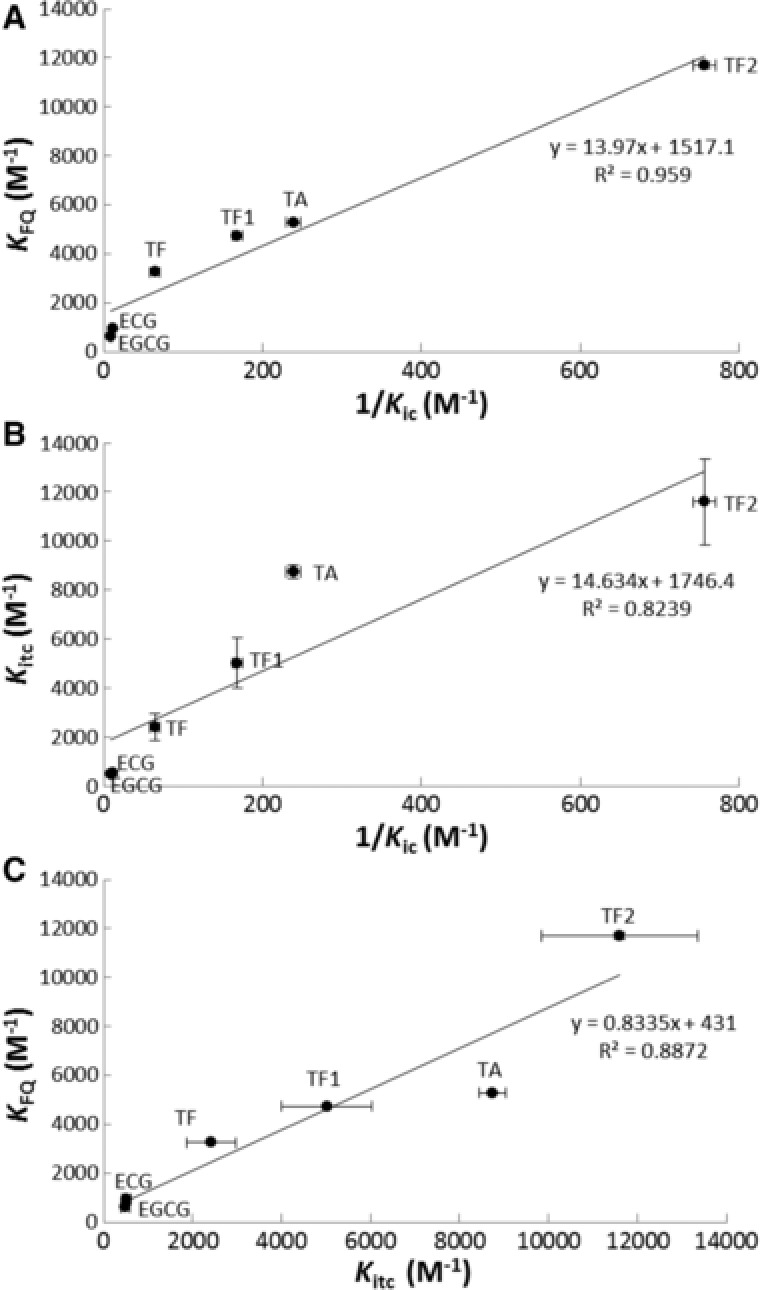
The linear correlations between *K*_FQ_ and 1/*K*_ic_ (**A**), *K*_itc_ and 1/*K*_ic_ (**B**) and *K*_FQ_ and *K*_itc_ (**C**). The respective correlation equations and coefficients (*R* ^2^) are listed as well.

The competitive inhibition constant, *K*_ic_, represents the dissociation constant of the PPA–polyphenol complex; therefore, the reciprocal of *K*_ic_ (1/*K*_ic_) indicates the association constant of polyphenols with PPA. As discussed above, quenching constant, *K*_FQ_, indicates the binding affinity of a quencher to protein; therefore, if the quencher binding is related to enzyme inhibition, there should be a relationship between *K*_FQ_ and 1/*K*_ic_. Based on this concept, the correlation between *K*_FQ_ and 1/*K*_ic_ is shown in Fig. 5A. There was a positive linear correlation between the two constants (*K*_FQ_ = 13.97·1/*K*_ic_ + 1517.1, *R* ^2 ^= 0.9590), suggesting that lower *K*_ic_ corresponds to higher *K*_FQ_. Hence, *K*_FQ_ and 1/*K*_ic_ obtained through fluorescence quenching and inhibition kinetics methods, respectively, may be combined to characterize the binding of polyphenols with PPA. FQ reflects the change of micro‐environment in the vicinity of Trp residues caused by both collisional encounters and complex formation between Trp and quenchers. In addition, other amino acid residues near Trp may also affect the fluorescence of a fluorophore 27. In contrast, 1/*K*_ic_ definitely reflects the formation of enzyme–inhibitor complex 6. Therefore, FQ can be more sensitive than inhibition kinetics in analysis of interactions between enzyme and polyphenol, which is reflected by the fact that a lower polyphenol concentration was needed to quench PPA fluorescence than to inhibit PPA activity. This may explain why the coefficient (the slope of the plot of *K*_FQ_ against 1/*K*_ic_) is much higher than 1. Previous studies also suggest that *K*_FQ_ values can be much higher than the inhibition constants 8, 31.

The dominant fluorophore of proteins is the indole group of the Trp residue. The indole aromatic heterocyclic residue shows a UV adsorption peak near 270 nm and a fluorescent emission peak near 340 nm. The emission wavelength of indole may be blue‐shifted (shorter wavelength) if the group is buried within a native protein, while it may be red‐shifted (longer wavelength) when the protein is unfolded 32. Therefore, the red‐shifted maximum *λ*_em_ of PPA by TEs, EGCG, ECG, TA and TF2 (Fig. 1A–C, E, F, H and I) indicates that partial structural unfolding occurs for PPA upon binding with these polyphenols. There was no evidence for structural unfolding of PPA bound with EC, EGC, TF1 or TF, as no significant red‐shifted maximum *λ*_em_ was observed (Fig. 1D, G, J and K).

The thermal stability of a protein is strongly dependent on its spatial structure, meaning that partial structural disruption for a protein may decrease its heat stability 33, 34. In this study, DSC was applied to provide supportive data for the potential structural unfolding of PPA indicated by red‐shifted maximum *λ*_em_. As shown in Table 1, TA induced 14 nm of red‐shift in maximum *λ*_em_ at its highest concentration. This was the largest shift for all the pure polyphenols investigated and was followed by ECG (9 nm), EGCG (6 nm) and TF2 (4 nm). This indicates that the order of the extent of potential structural unfolding for PPA upon binding with the four polyphenols was TA>ECG>EGCG>TF2. The denaturation process for a protein usually takes place in two steps. One is reversible, arising from the protein unfolding process. In this step, there is a partial loss of activity for the protein due to the disruption of intramolecular non‐covalent interactions 35, 36. The other one is irreversible, leading to the denaturation of the initially unfolded molecule 37. Therefore, the reversible unfolding process of an enzyme under external force (here, the interactions between polyphenols and PPA) is expected to promote the denaturation process of the enzyme during a DSC experiment. This was confirmed as TA which caused the most extensive PPA structural unfolding (reflected by the most red‐shift of maximum *λ*_em_), also made PPA most vulnerable to heat during DSC (reflected by the lowest denaturation temperature, *T*_d_, and energy required, *∆H*). Also, the *T*_d_ of PPA bound with ECG, EGCG, and TF2 corresponded to the extent of structural unfolding caused by binding of the three polyphenols, i.e. lower *T*_d_ corresponded to a higher red‐shift of maximum *λ*_em_. In addition, the *T*_d_ and *∆H* of PPA incubated with EGC, TF1, TF and EC remained similar to the PPA control, indicating that these four polyphenols did not change the thermostability of PPA. This is because they induced hardly any structural unfolding for PPA, since there was no significant red‐shift of maximum *λ*_em_ for PPA interacting with these polyphenols. Therefore, as no structural unfolding occurred, the thermostability of PPA did not change. A similar relationship of red‐shift of maximum *λ*_em_ and *T*_d_ (*∆H*) was also observed for PPA bound with three TEs, in which the higher mass ratio of TEs to protein caused lower thermostability of PPA. This is because higher concentration of TEs induced more extensive structural folding of the PPA molecule (Fig. 1A–C).

A similar observation has been reported for rice bran lipase, in which a decrease in apparent thermal denaturation temperature of the enzyme in the presence of chlorogenic acid and caffeic acid was observed 14. Some earlier studies also indicated that phenolic compounds decreased thermal stability of mono‐subunit and multi‐subunit proteins by binding with these proteins 15, 38, 39. Decrease in thermal stability of enzyme is usually associated with protein conformational changes. Tea polyphenols investigated in this study, as compounds with polyhydroxyl groups and aromatic rings in molecular structures, were able to interact with α‐amylase through both hydrogen bonding and hydrophobic forces 13, 31. This might induce conformational changes of PPA, leading to a shift in both intrinsic fluorescence and thermal stability of PPA. It should be noted that the potential unfolding of an enzyme by a phenolic compound is not necessarily related to its fluorescence quenching nor to enzyme inhibition. For example, both TF1 and TF could cause strong fluorescence quenching and inhibition of PPA, but no potential unfolding was observed by DSC thermograms for PPA bound with the two polyphenols.

The kinetics of inhibition of PPA indicates that TA, EGCG and TF2 were competitive inhibitors of PPA, specifically binding with the active site of the enzyme 6. In ITC binding analysis, the fitted single‐site binding model for the three polyphenols suggests that they were most likely to bind at a single set of binding sites. Therefore, the ITC results were consistent with the inhibition kinetics, in terms of specific binding sites on PPA for the competitive inhibitors. To further elucidate the correlation between the binding analyses by ITC and inhibition kinetics, the relationship between the binding constants obtained through the two methods were plotted (Fig. 5B). As shown, there was a positive linear correlation between *K*_itc_ and 1/*K*_ic_ (*K*_itc _= 14.634·1/*K*_ic _+ 1746.4, *R* ^2 ^= 0.8239), indicating that the equilibrium of competitive inhibition (suggested by 1/*K*_ic_) actually resulted from the binding of polyphenols with specific sites on the enzyme (suggested by *K*_itc_). Therefore, the constants obtained through the inhibition kinetics and ITC show a consistent relationship, and the two methods may be combined to analyze the binding of polyphenols with α‐amylase.

Very little corrected exothermic heat was observed for the titration of EC and EGC into PPA (Fig. 4G and H), indicating that non‐galloylatedcatechins hardly interacted with PPA. This is in agreement with the very weak binding of non‐galloylatedcatechins with PPA that was demonstrated by the low inhibition and fluorescence quenching of PPA by EC and EGC (Table S1 and Fig. 1D and G). By introduction of a galloyl group, EC and EGC were transformed to their galloylated forms, ECG and EGCG, respectively (Supporting Information Fig. S1). Then, the binding of ECG and EGCG with PPA could be detected by the corrected heat released for the respective titration of the two polyphenols into PPA. Similarly, for the theaflavin family, TF1 and TF2 were the mono‐ and di‐galloylated forms of TF (without a galloyl group in its molecule), respectively (Supporting Information Fig. S1), and both the *∆H* and *K*_itc_ values of the three compounds were in the order of TF2>TF1>TF. Therefore, for catechin and theaflavin families, the introduction of galloyl groups in the molecular structures could enhance the binding of these polyphenols with PPA. Additionally, TA, with ten galloyl groups in its molecular structure had a high binding affinity to PPA (*K*_itc_, 8740 M^−1^; *∆H*, −7273 J mol^−1^). This finding is consistent with our previous results that galloyl substitution plays an important role in association of catechins and theaflavins with PPA, as deduced from the kinetics of inhibition of PPA by tea polyphenols 6. Interestingly, the role of galloyl moiety in binding with PPA is also reflected by the apparent stoichiometry (*n*), i.e. the polyphenol to PPA ratio 17. Tannic acid which has the most galloyl groups in all the polyphenols investigated, had the lowest stoichiometry (*n*, 4), indicating that the fewest TA molecules are needed to saturate the binding with PPA. In addition, as the number of galloyl moieties decreased in the theaflavin molecules, *n* increased (*n* for TF2, TF1 and TF were 15, 17 and 24, respectively). The changing tendencies of *n* for both ECG to EC and EGCG to EGC were the same as that for theaflavins. Through molecular docking analysis it appeared that the galloyl moiety interacts with PPA, not only through hydrogen bonds between its three hydroxyl groups and the catalytic amino acid side‐chains (Asp197, Glu233 and Asp300), but also through hydrophobic π‐π (aromatic‐aromatic) interactions with the active site of the enzyme (Trp59) 4, 5. Therefore, based on our results, it is likely that the galloyl moiety in tea polyphenols binds with PPA and further promotes polyphenols entering and associating with the active site of the enzyme.

As both *K*_FQ_ and *K*_itc_ demonstrate the binding of polyphenols with PPA, it is of interest to analyze the correlation between the two constants in order to evaluate the accuracy of each method and the rationality of combining fluorescence quenching and ITC. As shown in Fig. 5C, there was a positive linear relationship between *K*_FQ_ and *K*_itc_ (*K*_FQ _= 0.8335*K*_itc_ + 431, *R* ^2 ^= 0.8872). Because both constants directly reflected the interactions between polyphenols and PPA, the correlation coefficient was close to 1. This also indicates that the binding constants obtained through fluorescence quenching and ITC are comparable and that analysis of the interactions between polyphenols and enzyme by combination of the two methods is feasible. In addition, the fact that *K*_FQ_ and *K*_itc_ are quantitatively similar suggests that tea polyphenols binding to PPA manly include the interactions with the tryptophan and with the vicinity of tryptophan at the active sites of the enzyme.

## 5 Conclusion

Overall, our data show that tea polyphenols which had higher inhibitory effects on PPA showed higher fluorescence quenching effects on the enzyme. Our study shows, for the first time, the potential structural unfolding of PPA upon binding of polyphenols with the enzyme as indicated by the red‐shift of maximum *λ*_em_ in FQ and the decreased thermostability of PPA in DSC. Through ITC analysis and inhibition kinetics, it was shown that the equilibrium of competitive inhibition resulted from the binding of polyphenols with specific sites on the enzyme. There were positive and linear correlations among 1/*K*_ic_, *K*_FQ_ and *K*_itc_, indicating that all three constants reflect the binding affinity of polyphenols to α‐amylase. The galloyl moiety was shown to be an important substituent group in the binding of catechins and theaflavins with α‐amylase and thus in inhibiting the catalytic activity of the enzyme; therefore, galloyl substitution should be considered in the extraction and synthesis of pharmaceutical ingredients for type II diabetes.

## Supporting information

Supplementary information

Table S1. Kinetics of PPA inhibition by TEs and pure phenolic compounds [6].
