# Supplementary figures and images for: The mechanism of interactions between tea polyphenols and porcine pancreatic alpha‐amylase: Analysis by inhibition kinetics, fluorescence quenching, differential scanning calorimetry and isothermal titration calorimetry
Source: Mol Nutr Food Res. 2017 Aug 23;61(10):1700324. doi: 10.1002/mnfr.201700324 (PMC5656823; doi:10.1002/mnfr.201700324)

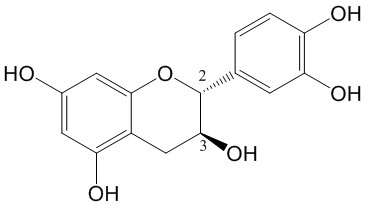


**A**

**C**

**B**

**(C)**


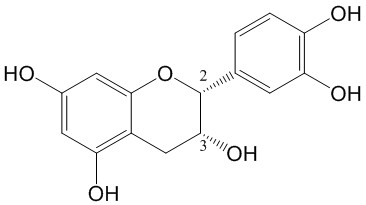


**A**

**C**

**B**

**(EC)**


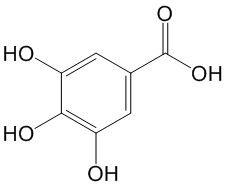

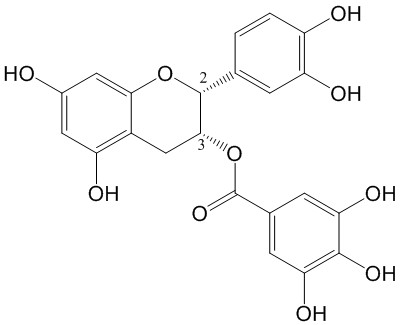


**A**

**C**

**B**

**(ECG)**


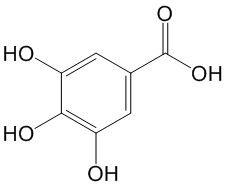

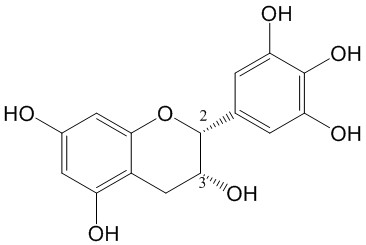


**A**

**C**

**B**

**(EGC)**


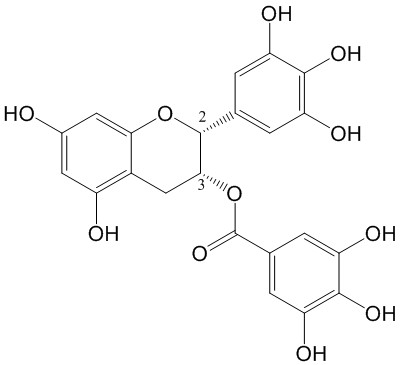


**(EGCG)**

**A**

**C**

**B**


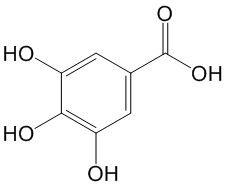

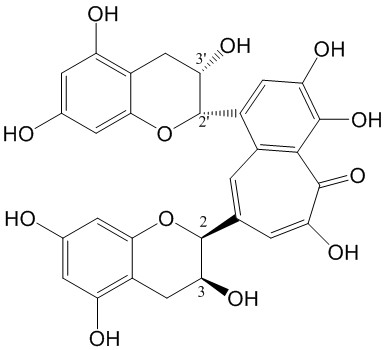


**A**

**C**

**A’**

**C’**

**(TF)**


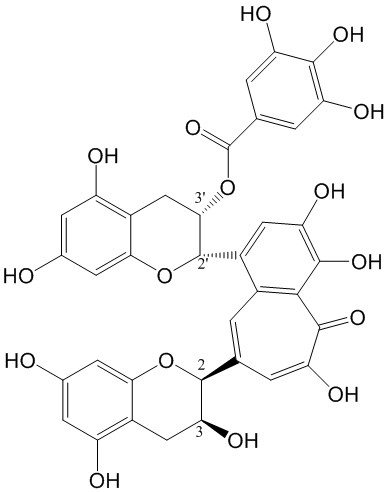


**(TF1)**

**A**

**C**

**A’**

**C’**


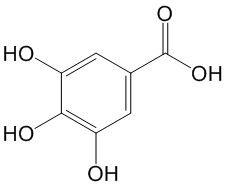

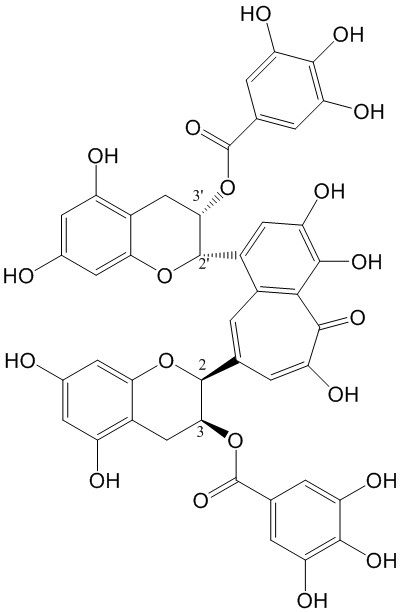


**A’**

**C’**

**A**

**C**

**(TF2)**

**(Galloyl)**

**(Galloyl)**

**(Galloyl)**

**(Galloyl)**


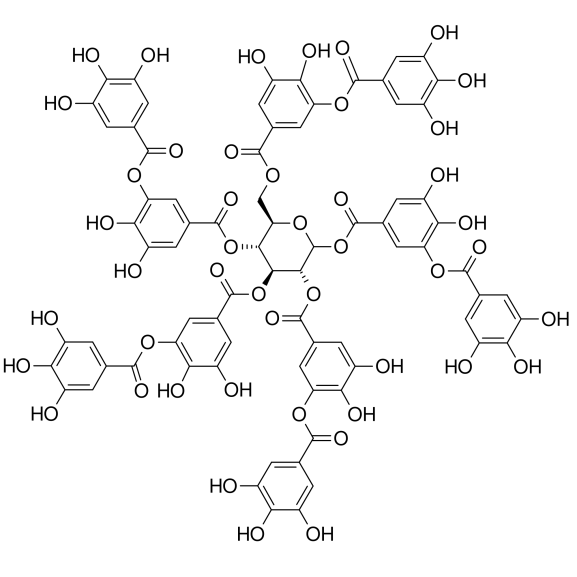


**(TA)**

**3’’**

**3’’**

**3’’**

**3’’**

**3’’**

Supplement: Supplementary file 1 — Supplementary information [file MNFR-61-na-s001.docx]
