# Supplementary material for: The mechanism of interactions between tea polyphenols and porcine pancreatic alpha‐amylase: Analysis by inhibition kinetics, fluorescence quenching, differential scanning calorimetry and isothermal titration calorimetry
Source: Mol Nutr Food Res. 2017 Aug 23;61(10):1700324. doi: 10.1002/mnfr.201700324 (PMC5656823; doi:10.1002/mnfr.201700324)
Supplement: Supplementary file 2 — Table S1. Kinetics of PPA inhibition by TEs and pure phenolic compounds [6]. [file MNFR-61-na-s002.docx]

| Phenolics | Parameters of kinetics of inhibition | | | | |
| --- | --- | --- | --- | --- | --- |
|  | *K*_ic_ (mon/L) | *K*_iu_ (mol/L) | 1/*K*_ic_ (L/mol) | 1/*K*_iu_ (L/mol) | IC_50_ (mg/mL) |
| GTE | 6.332 (mg/mL)^A^ | NA | 0.158 (mL/mg)^C^ | NA | 0.197^A^ |
| BTE | 7.847 (mg/mL)^B^ | NA | 0.127 (mL/mg)^B^ | NA | 0.462^B^ |
| OTE | 17.122 (mg/mL)^C^ | NA | 0.058 (mL/mg)^A^ | NA | 0.721^C^ |
| EGCG | 0.104^f^ | NA | 9.611^a^ | NA | 2.514^f^ |
| ECG | 0.085^e^ | 0.101^c^ | 11.798^b^ | 9.926^a^ | 1.729^e^ |
| TF2 | 0.001^a^ | NA | 756.716^f^ | NA | 0.130^a^ |
| TF1 | 0.006^c^ | 0.028^a^ | 168.414^d^ | 35.965^c^ | 0.244^b^ |
| TF | 0.015^d^ | 0.056^b^ | 64.773^c^ | 18.004^b^ | 0.412^d^ |
| TA | 0.004^b^ | NA | 238.797^e^ | NA | 0.301^c^ |
| EC | - | - | - | - | 9.321^g^ |
| EGC | - | - | - | - | 14.125^h^ |

**Table S1.** Kinetics of PPA inhibition by TEs and pure phenolic compounds [6].

^*^ Different letters in the same column represent significantly different mean values (*P*<0.05). ‘NA’, not available. ‘-’, not detected due to very weak inhibition by EC and EGC.
